# Electronic health records reveal that COVID-19 impacted health resources and survival of Basque population

**DOI:** 10.1007/s40520-024-02884-7

**Published:** 2024-11-29

**Authors:** Sara Cruces-Salguero, Igor Larrañaga, Javier Mar, Ander Matheu

**Affiliations:** 1 Cellular Oncology group, Biodonostia Health Research Institute, Paseo Dr. Beguiristain s/n, 20014 San Sebastian, Spain; 2 Osakidetza Basque Health Service, Debagoiena Integrated Healthcare Organisation, Research Unit, Mondragón, Gipuzkoa Spain; 3 Kronikgune Institute for Health Services Research, Barakaldo, Spain; 4 Biodonostia Health Research Institute, San Sebastian, Gipuzkoa, Spain; 5 IKERBASQUE, Basque Foundation for Science, Bilbao, Spain; 6 Centro de Investigación Biomédica en Red de Fragilidad y Envejecimiento (CIBERfes), Carlos III Institute, Madrid, Spain

**Keywords:** Pandemic, Public health, Electronic health records

## Abstract

**Background:**

The COVID-19 pandemic impacted worldwide. The Basque Country was one of the regions in Spain most affected by the virus.

**Methods:**

In this retrospective study, we took advantage of the Basque Health Service electronic health records data lake of over 20,000 deceased individuals, including 5000 positives for COVID-19, between 2020 and 2022 in Gipuzkoa (Basque Country, Spain).

**Results:**

Comparison between COVID-19-positive and negative individuals’ showed that the prevalence of infections was higher inside nursing homes and COVID-19 promoted a significant rise in hospitalizations, emergency entrances, and ICU admissions. No differences were observed between genders in terms of infections or survival but were detected in health resources and vaccination showed a strong protective effect against the disease.

**Conclusions:**

Our results provided a complete characterization of the impact of COVID-19 on the Basque population, which expands the knowledge of the pandemic on older individuals and the health system. Our study also highlights the benefit of the use of Electronic Health Records in studying human diseases.

**Supplementary Information:**

The online version contains supplementary material available at 10.1007/s40520-024-02884-7.

## Introduction

In March 2020, the coronavirus disease 2019 (COVID-19) was declared a pandemic by the World Health Organization (WHO) [1]. Since the pandemic started, over 750 million cases of COVID-19 have been reported, and more than 7 million people have died from the disease [2]. Since the beginning, multiple epidemiological and clinical studies tried to discern the general impact on the population of different regions and worldwide as well as the consequences and mechanisms behind COVID-19 effect. Regarding the impact, the clinical profile presented by COVID-19 varies between individuals. Most infected people manifest an asymptomatic condition or experience mild or moderate respiratory symptoms [3]. However, vulnerable populations, such as older people and those with previous comorbidities and higher degrees of frailty, are at risk of presenting severe complications, which can finally lead to death [4]. Indeed, age was identified as an independent risk factor for death and contributed to the greater severity of COVID-19 [5].

Remarkably, most of the current literature regarding COVID-19 was published during the first two years of the pandemic [6–8] and, in particular, most of the studies on the older population were conducted on one specific, selected and limited patient cohort. They typically relied on cases and mortality statistics, while others focused on groups already suffering from specific diseases. However, it is known that the repercussions of COVID-19 concerning public health changed during the whole pandemic period, due to factors such as the variable healthcare load across the different waves [9], the virulence of the virus variants [10] or the start and number of doses of vaccination [11]. Therefore, very few studies took into account the combination of all of these variables when analyzing the impact of COVID-19 on the older population. It is yet uncertain how each pandemic wave and the predominant strains contribute to varying outcomes in the older population with controversial results in terms of gender [6, 12], functional capacity [13–15] and clinical impact [16–18]. In this sense, the use of the information recorded in health services, such as Electronic Health Records (EHR), provide a complete overview of the impact of the COVID-19 for each individual in their particular context [19].

The statistics situated Spain among the most affected countries by COVID-19 in Europe, both in terms of total cases and in deaths per million people [20]. Within Spain, the Basque Country was one of the regions most impacted. The first case was detected on the 28^th^ of February 2020, and since then, there have been over 800,000 infections and 8,000 deaths [21]. The region of the Basque Country is one of the most aged from Spain and Europe, and this population aging has been associated with multimorbidity of chronic diseases whose prevalence increases along with age [22], therefore representing an especially vulnerable population for COVID-19 disease. In fact, an observational study identified the variables associated with COVID-19 severity within the general population and revealed that older cases were more likely to experience severe outcomes [23]. However, a previous study from the group also showed that the centenarian population of the Basque Country, who had been previously characterized presenting a lower number of aging-associated diseases, comorbidities and reduced need for clinical resources [24], was more resilient to COVID-19 than other old population groups [25]. Moreover, Basque Country have been considered a relatively isolated region, being Basques one of the most ancient European populations and maintaining some genetic characteristics. Because of all of this, a deeper characterization of the impact of COVID-19 on the Basque Country’s older adults might help in understanding the detailed response of the aged population against the disease. For that, we took advantage of EHR data from Basque Health Service, Osakidetza, which follows a National Health System (NHS) Beveridge type system and has high quality performance indicators [26], a key aspect to develop effective public health responses to the pandemic [27, 28]. In this work, we described and analyzed the effect COVID-19 had on the Basque old population, in terms of infections, derived clinical conditions, use of resources, and survival taking into account the different COVID-19 pandemic waves.

## Materials and methods

### Design

An observational study was carried out with Real-World Data, retrospectively analyzing the impact of COVID-19 on the Basque population. The differences in the impact of the disease were measured in terms of the number of positive tests, levels of independence according to Barthel index, diagnoses, operations, pharmacological treatments, the use of contacts with the health system, and survival after COVID-19.

### Study population

As in previous works from the group [25], this study was based on deceased individuals between 28^th^ February 2020 and 31^st^ December 2022 recorded in the data lake of the Basque Health Service. A data lake is a collection of various data assets that are stored within a Hadoop ecosystem with minimal change to the original format or content of the source data. The information registered in the database includes patient-level demographic and clinical data and it was anonymized previously. Only with the identifiers provided to the researchers, the identity of any patient cannot be known or traced.

First, the quality of the data was evaluated to delete those patients whose records did not fulfill the quality criteria. If record of date of birth and/or death did not have the appropriate format (dd/mm/yyyy) or had missing values, if date of birth was posterior to the date of death or if there was more than one date of death, those patients were excluded.

21,495 individuals remained after this data cleansing (Supplementary Figure 1A). The database included, for each patient, demographic information such as sex, age, province, and status in nursing home, and clinical information such as diagnoses, use of medical resources, prescription of drugs, levels of independence, and records of COVID-19 tests.

Regarding diagnoses, the information included all records of episodes of primary care, emergency, outpatient, and in-hospital care and was recorded through the International Classification of Diseases, Ninth Revision (ICD-9), and Tenth Revision (ICD-10) diagnosis codes. Clinical resources information included visits to Primary Care Nurse, Primary Care Physician, Outpatients, Home Hospitalization, Hospitalization, Procedures, Intensive Care Unit (ICU), and Emergencies. Pharmaceutic prescription information was classified according to the Anatomical Therapeutic Chemical (ATC) code.

Some individuals had records of levels of independence, measured through the Barthel index, which assesses the ability of individuals performing activities of daily living [29]. A Barthel index of one represented total dependency, and five, independency.

COVID-19 infections were confirmed by tests performed by the Basque Health Service. These tests could be asked by the different assistance areas: Primary Care, Outpatients, Hospitalizations, Home Hospitalizations, and Emergencies. 5072 patients (23.6%) had a positive COVID-19 test. 3249 patients (15.12%) never had a COVID-19 test and therefore were considered negative, with a total of 16,423 patients (76.4%) negative for COVID-19. Even though data about vaccination was not available in the database, according to the Basque Health Department [30], vaccination in the Basque Country started on the 27^th^ of December 2020, and more than 90% of Basque individuals above 50 years was considered vaccinated on the 16^th^ of August 2021. Those dates were taken as reference for assessing vaccination status of individuals.

### Statistical analysis

For comparisons of the demographic data between COVID-19 positive and negative individuals, Pearson’s chi-square test was used. A two-sided *p*-value of < 0.05 was considered statistically significant.

Survival of individuals was assessed through Kaplan-Meier estimator and Cox regression. In this analysis, only individuals positive for COVID-19 were studied (n = 5072). Individuals with COVID-19 detected after decease (n = 12) were excluded (final n = 5060). We studied overall survival from COVID-19 detection until decease of the patients. “Death because of COVID-19” was defined as those deceases that happened until 30 days after COVID-19 detection, based on World Health Organization (WHO) data as we did in previous studies [31]. The rest of individuals were treated as censored data since they were considered “survivors” for COVID-19. In the Cox regression, we analyzed the impact on survival of different ages, gender, status in nursing home, Barthel index and its interaction with sex, and vaccination of patients. Since nursing homes were a focus of infections during COVID-19 pandemic, we also took into account its interaction with age in this analysis.

We took clinical records from the pandemic onward for analyzing COVID-19 impact. Tests of difference in means were applied to random subsampling (300 COVID- versus 100 COVID+) independently repeated 100 times, due to the big sample size leading to very small *p*-values. Shapiro-Wilk test was applied for assessing normality, and Student’s t-test or Mann-Whitney U test was employed depending on the result. The resulting *p*-values were corrected using the False Discovery Rate (FDR), and the median *p*-value was presented. In the case of the use of resources, only the population outside nursing homes was considered, for avoiding potential bias.

The building and transformation of the database were performed in Python 3.9. All the statistical analyses were performed in RStudio, v.4.2.2 (URL: https://www.r-project.org/). Survival [32] and survminer packages were used for survival analysis.

### Ethics

This study was approved by the Basque Clinical Research Ethics Committee (CEIm-E code PI2020206) and adhered to the tenets of the Declaration of Helsinki by the World Medical Association regarding human experimentation.

## Results

### Epidemiological analysis

**Fig. 1:**
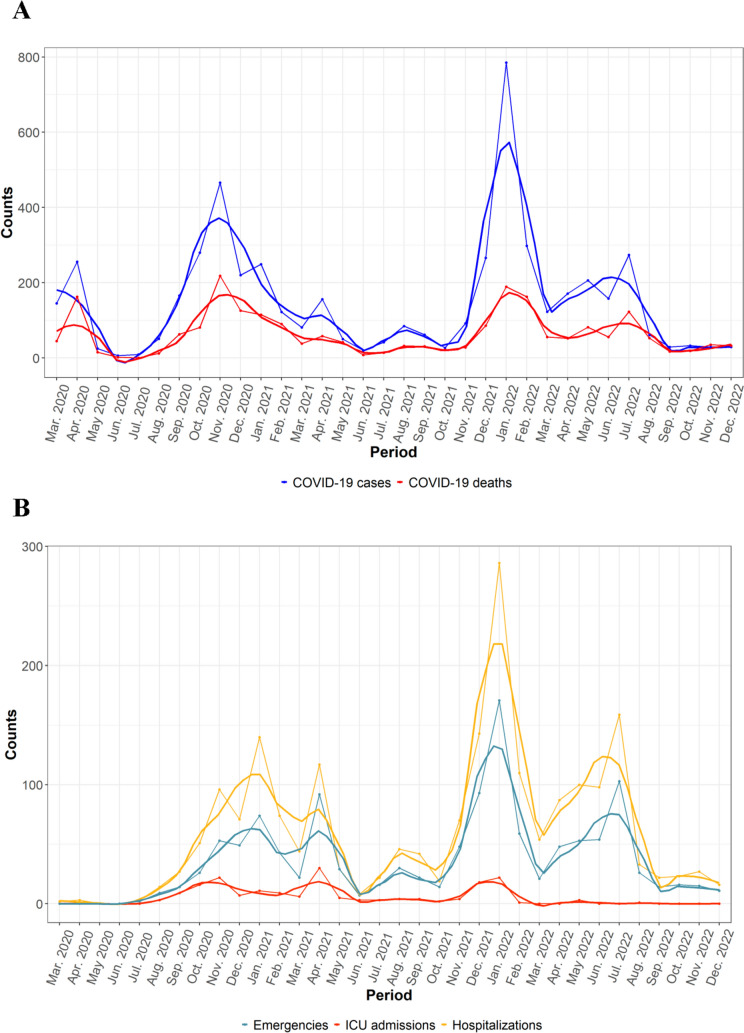
Evolution of COVID-19 pandemic. **A** Evolution of COVID-19-positive cases and deaths during the pandemic. **B** Evolution of the use of hospitalizations, emergencies and ICU admissions during the pandemic. Only records that had a COVID-19 diagnosis associated were considered

We studied a population of 21,495 individuals deceased in Gipuzkoa (region of the Basque Country) since the first COVID-19 case was detected on the 28^th^ of February 2020 until the 31^st^ of December 2022, five months before the World Health Organization declared the end of the pandemic [33] (Supplementary Figure 1B). During the studied period, a total of 5072 (23.6%) individuals were infected with the SARS-CoV-2 virus, while 16,423 (76.4%) were not. We found that women and men got infected equally, with 2638 (52%) and 2434 (48%) of positive cases, respectively. We saw notable peaks of infections in March 2020 (beginning of the pandemic), in Autumn 2020, and in January 2022, corresponding to the dates in which a higher number of cases were detected in the Basque Country, according to the Basque Health Department [34]. The highest peak of infections was detected in January 2022, with almost 800 cases recorded. From September 2022 onward, the incidence of COVID-19-positive individuals notably decreased (Fig. 1A). No differences between women and men in number of infections were observed in the different COVID-19 waves (Supplementary Figure 2A). When assessing demographic variables, there was a higher proportion of infections in nursing homes than outside, with 30% of COVID-19-positive individuals being residents (*p* = 0.003). However, we did not find differences in infections related to the levels of independence of individuals, measured through the Barthel index **(**Table 1**).**

**Table 1:** Demographic features of COVID+ and COVID− population

	COVID+ (n = 5072)	COVID− (n = 16,423)	*p*-value
Demographic data			
Sex	2638 women (52.01%) 2434 men (47.99%)	8110 women (49.38%) 8313 men (50.62%)	0.966
Residence	3530 no (69.6%) 1542 yes (30.4%)	14,181 no (86.35%) 2242 yes (13.65%)	**0.003**
Functional data			
Barthel index	1031 independent (20.33%) 1394 minimally dependent (27.48%) 478 partially dependent (9.42%) 385 very dependent (7.59%) 499 total dependent (9.84%)	3373 independent (20.54%) 4076 minimally dependent (24.82%) 1385 partially dependent (8.43%) 1118 very dependent (6.81%) 1623 total dependent (9.88%)	0.915

Number of individuals and percentages (%). *p*-value of Pearson’s chi-square test between COVID-19 infection status and demographic variables

Significant *p*-values (< 0.05) are displayed in bold

### Clinical analysis

Next, we analyzed the impact of COVID-19 on the Basque population, taking advantage of a large battery of clinical information included in the Osakidetza data lake. We observed that COVID-19-positive individuals had a higher number of respiratory system-, nervous system- and circulatory system-related diagnoses, as well as a higher number of symptoms, signs, and ill-defined conditions, indicating that infected individuals had more pathologies after COVID-19 infection **(**Table 2 and Supplementary Table 1). Regarding operations, those infected with COVID-19 had a similar number of a wide range of them but a significantly higher number of miscellaneous diagnostic and therapeutic procedures, category which included mechanical ventilation, among others (*p* = 0.015) **(**Table 2 and Supplementary Table 2). In the case of pharmacological treatment, we saw a significant increase in the prescription of blood-related drugs and anti-infectives for systemic use in COVID-19-positive individuals and an overall higher prescription of drugs, displaying an increased need for drugs since the pandemic started (*p* < 0.001) (Table 2 and Supplementary Table 3). However, the most notable rises were found in the use of medical resources, with almost all of them significantly increased in COVID-19-positive individuals **(**Table 2**)**. Particularly, the number of hospitalizations had a mean increase of almost 10 hospitalizations when compared to non-infected people (*p* < 0.001), and the use of emergencies displayed a mean of 4 entrances in comparison with 2 in the case of COVID-19-negative individuals (*p* < 0.001). Finally, in the case of intensive care unit (ICU) attendance, which was particularly increased in the pandemic context, COVID-19-positive individuals showed a significant increase when compared to the non-infected population, with each COVID-19 patient staying a mean of 2 days **(**Table 2**).** All these numbers indicated a higher need for medical resources from the infected population. Furthermore, when performing the same analysis divided by sex, we obtained similar results, with significant increases in almost all areas but particularly in hospitalizations and emergencies for both women and men. Interestingly, only COVID-19-positive men displayed a significant rise in ICU attendances (*p* = 0.023), spending a mean of almost 3 days while women stayed a mean of 1 day in ICU. Remarkably, COVID-19-positive men also had a notably higher mean of hospitalizations than women (28 vs 20) (Supplementary Table 4).

**Table 2:** Analysis of clinical history of patients in COVID-19 contex

Variable	COVID+	COVID−	*p*-value
Diagnoses			
Diseases of the nervous system and sense organs	1.37 ± 2.09	0.96 ± 1.74	**0.049**
Diseases of the circulatory system	4.58 ± 6.9	3.31 ± 5.4	0.050
Diseases of the respiratory system	1.91 ± 3.18	1.05 ± 2.39	**< 0.001**
Symptoms, signs, and Ill-defined conditions	3.68 ± 3.22	1.68 ± 2.1	**< 0.001**
Operations			
Miscellaneous diagnostic and therapeutic procedures	2.27 ± 3.88	1.35 ± 2.7	**0.015**
Drugs			
Blood and blood forming organs drugs	1.32 ± 1.97	0.86 ± 1.53	**0.036**
Antiinfectives for systemic Use	2.97 ± 3.67	1.83 ± 2.74	**0.001**
Total number of drugs	19.45 ± 16.32	13.36 ± 12.81	**< 0.001**
Use of resources			
Primary care nurse	22.05 ± 28.07	16.22 ± 24.12	**0.001**
Primary care physician	22.93 ± 19.66	16.42 ± 16.02	**< 0.001**
Outpatients	9.32 ± 15.86	7.26 ± 13.19	0.836
Home hospitalizations	3.07 ± 10.01	2.5 ± 7.84	0.68
Hospitalizations	24.43 ± 26.86	15.45 ± 20.38	**< 0.001**
ICU	0.11 ± 0.35	0.05 ± 0.24	**0.042**
Days at ICU	1.92 ± 8.59	0.36 ± 3.24	**0.036**
Emergencies	3.76 ± 4.21	2.31 ± 3.05	**< 0.001**

Mean ± SD. *p*-value of test of difference in means of COVID-19- positive vs negative individuals

Significant *p*-values (< 0.05) are displayed in bold

We observed that the evolution of the use of medical resources during the pandemic was similar to the evolution of the number of infections, with the peaks in the number of positives correlating with peaks in hospitalizations, emergencies, and ICU **(**Figs. 1A, B**).** Notably, there was barely no increase in the records of clinical resources during the first months of the pandemic, even though hospitalizations and ICU entrances were included in the Basque Health Department official reports [35].

### Survival analysis

**Fig. 2:**
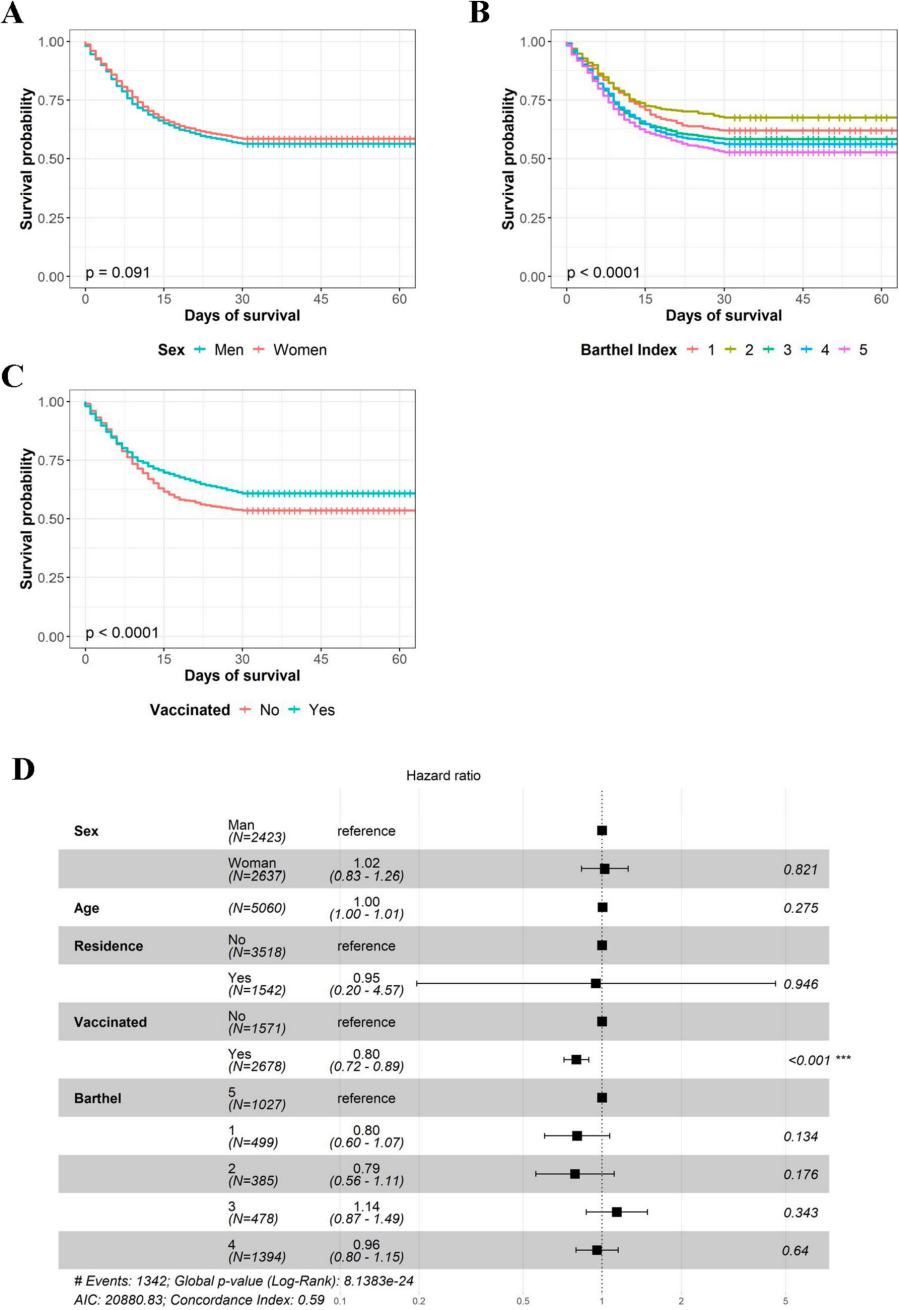
Survival analysis of COVID-19 30-day mortality. **A** Survival of men vs women. **B** Survival in function of Barthel index, from 1 (totally dependent) to 5 (independent). **C** Survival of vaccinated vs non-vaccinated individuals. **D** Cox regression of the main demographic variables

Finally, we analyzed the survival of the individuals facing SARS-CoV-2 infection. When comparing sex, we did not detect significant differences when assessing overall survival but found a slight tendency of women exhibiting extended survival at 30 days **(**Fig. 2A and Supplementary Figure 2B). On the other hand, we observed that individuals with lower levels of independence exhibited a more extended survival than the independent ones, both at 30 days and at overall survival. **(**Fig. 2B and Supplementary Figure 2C). Next, knowing that the process of vaccination completely changed the response of patients to COVID-19 [36], we took this factor into account in the analysis. Thus, vaccinated individuals lived more when compared to individuals infected during the first waves of the pandemic (*p* < 0.001), and this effect remained in the Cox regression even when adjusted by other demographic variables **(**Fig. 2C, D**)**. However, sex and Barthel index had no significant effect in the survival of COVID-19 patients after taking into account the rest of the variables. Finally, when assessing the evolution of deaths across the whole study period, we saw that April 2020, November 2020, and January 2022 were the months with more deceases recorded. Interestingly, during the first year of the pandemic, infections and deaths followed a similar trend, but from January 2022 onward, the recorded peak in the number of infections was not accompanied by a proportional peak in the number of deaths, which could be attributed to the protective effect of vaccination **(**Fig. 1A**)**.

## Discussion

The COVID-19 pandemic affected a high proportion of the population and implied an increased load on the healthcare system. Consistent with previous reports [37, 38], our demographic data showed that the incidence of COVID-19 infections was higher in nursing homes. We also found that higher levels of dependency were associated with extended survival, which did not fit the findings of previous studies using smaller cohorts, in which a lower Barthel index was associated with higher mortality in COVID-19 patients [14, 15]. However, it must be considered that all patients in our cohort were deceased, and among them, it could be expected that most dependent individuals had more surveillance and faster contact with health services. Furthermore, this protective effect was removed when performing the multivariate regression and adjusting by other variables, which suggests a potential relation between dependency status and other confounders. Interestingly, previous works from the group showed that centenarians, who exhibited higher levels of dependence [24], had lower hazard ratios in COVID-19 survival when compared to younger population groups [25].

Regarding gender, we did not find differences in terms of infections in women and men, which agrees with the findings of other studies [39]; however, higher risk of mortality and severity of the disease has been regularly associated with male gender [8, 12, 39, 40], which has been associated with differences in immune response and sex-specific comorbidities [41]. We also found this tendency, with men exhibiting poorer survival at 30-days analysis. Furthermore, COVID-19-positive men showed a stronger need of clinical resources, displaying increased numbers of ICU attendances and days, hospitalizations, and emergencies when compared to women, which suggests more severe clinical outcomes. On the other hand, the strong protective effect of vaccination was undeniably demonstrated in the survival analysis, which other authors had confirmed with a lower risk of ICU admissions [42] and COVID-19-derived cardiopathies in vaccinated individuals [43].

The analysis of the clinical history of patients manifested the impact of COVID-19. Infected individuals had more diseases, more diagnostic and therapeutic interventions, and were prescribed more drugs since the pandemic started, indicating an overall higher vulnerability when compared to the non-infected population. In fact, previous studies focused on specific comorbidities identified muscle weakness, cerebrovascular accidents, or acute respiratory failure, among others, as signs of severe COVID-19 or long-term COVID-19 [44, 45], symptoms that agree with our findings. Finally, the analysis of clinical resources pointed out the repercussions of the disease: almost all resources were increased in infected individuals. Particularly, hospitalizations, emergencies, and ICU admissions displayed a dramatic rise, following the tendency exhibited in other studies that analyzed 2020-2021 period [46]. All in all, the use of EHR data in a large cohort of older patients provided an enriched overview of the COVID-19 impact of different aspects on the Basque population, as shown in another study that followed similar approaches [19].

This study has some limitations. The lack of information regarding the specific cause of death of each individual could be distorting the survival analysis. While data about the real vaccination status was missing, we performed an approximation based on official reports [30]. On the other hand, we took the last recorded functional measurement of individuals when assessing Barthel index, which could have been recorded far after or before COVID-19 infection and therefore would not be completely accurate. In addition, we did not take into account other factors such as comorbidities or polypharmacy, which could also be acting as cofounders. Regarding the analysis of the impact, even though we only took records pandemic onward, the association of medical conditions and use of resources with COVID-19 cases could not be demonstrated. Moreover, during the first months of the pandemic, COVID-19-associated hospitalizations and ICU entrances were still not recorded in the EHR, therefore clinical records from this first period might be missing.

In summary, we have characterized the main aspects resulting from the COVID-19 pandemic in the Basque Country. Our results showed that COVID-19 had a great impact on the old Basque population, with increases in diagnoses, drugs, procedures, and particularly, clinical resources, and highlighted the benefits of vaccination. While no significant differences were found regarding infections and survival, men seemed to be more vulnerable to the disease with a higher need of clinical resources. These data enriched the information on COVID-19 in the aged population and confirmed the notable increase in clinical resources, particularly during the first periods of the pandemic. Moreover, our study reinforces the usefulness of individual-based approaches and the benefit of the use of Electronic Health Records in studying human trajectories and diseases.

## Supplementary Information

Below is the link to the electronic supplementary material.

Supplementary file1 (DOCX 4194 KB)

Supplementary file2 (PPTX 3104 KB)

## Data Availability

“The authors confirm that the data supporting the findings of this study are available within the article and its supplementary materials. The data that support the findings of this study are available from Basque Health Service but restrictions apply to the availability of these data, which were used under license for the current study, and so are not publicly available. Data are however available from the authors upon reasonable request and with permission of Basque Health Service.”
