# Supplementary material for: Electronic health records reveal that COVID-19 impacted health resources and survival of Basque population
Source: Aging Clin Exp Res. 2024 Nov 29;36(1):228. doi: 10.1007/s40520-024-02884-7 (PMC11606984; doi:10.1007/s40520-024-02884-7)
Supplement: Supplementary file 1 — Supplementary file1 (DOCX 4194 KB) [file 40520_2024_2884_MOESM1_ESM.docx]

Supplementary Table 1

Supplementary Table 2

Supplementary Table 3

Supplementary Table 4

Supplementary Figure 1

Supplementary Figure 2

**Supplementary Table 1.** Recorded diagnoses in COVID-19 context. Mean ± SD. p-value of test of difference in means of COVID-19 positive vs negative individuals.

| ***Diagnoses*** | **COVID+** | **COVID-** | ***p*-value** |
| --- | --- | --- | --- |
| Infection and Parasitic Diseases | 0.11 ± 0.45 | 0.06 ± 0.3 | 0.238 |
| Neoplasms | 2.25 ± 5.96 | 2.45 ± 5.53 | 0.966 |
| Endocrine, Nutritional and Metabolic Diseases, and Immunity Disorders | 2.19 ± 3.78 | 1.54 ± 3.02 | 0.127 |
| Diseases of Blood and Blood Forming Organs | 0.6 ± 1.41 | 0.47 ± 1.23 | 0.717 |
| Mental Disorders | 0.9 ± 1.82 | 0.62 ± 1.48 | 0.153 |
| Diseases of the Nervous System and Sense Organs | 1.37 ± 2.09 | 0.96 ± 1.74 | **0.049** |
| Diseases of the Circulatory System | 4.58 ± 6.9 | 3.31 ± 5.4 | 0.050 |
| Diseases of the Respiratory System | 1.91 ± 3.18 | 1.05 ± 2.39 | **< 0.001** |
| Diseases of the Digestive System | 0.97 ± 2.31 | 0.73 ± 1.77 | 0.460 |
| Diseases of the Genitourinary System | 1.54 ± 2.84 | 1.02 ± 2.07 | 0.088 |
| Complications of Pregnancy, Childbirth, and the Puerperium | 0 ± 0 | 0 ± 0.01 | - |
| Diseases of the Skin and Subcutaneous Tissue | 0.61 ± 1.84 | 0.39 ± 1.16 | 0.197 |
| Diseases of the Musculoskeletal System and Connective Tissue | 0.66 ± 1.53 | 0.46 ± 1.11 | 0.575 |
| Congenital Anomalies | 0.02 ± 0.23 | 0.02 ± 0.26 | - |
| Certain Conditions Originating in the Perinatal Period | 0 ± 0.04 | 0 ± 0.02 | - |
| Symptoms, Signs, and Ill-defined Conditions | 3.68 ± 3.22 | 1.68 ± 2.1 | **< 0.001** |
| Injury and Poisoning | 1.13 ± 1.93 | 0.82 ± 1.63 | 0.287 |
| Supplementary Factors Influencing Health Status and Contact with Health Services | 0.02 ± 0.15 | 0.01 ± 0.12 | 0.747 |

**Supplementary Table 2.** Operations in COVID-19 context. Mean ± SD. p-value of test of difference in means of COVID-19 positive vs negative individuals.

| ***Operations*** | **COVID+** | **COVID-** | ***p*-value** |
| --- | --- | --- | --- |
| Operations on the Eye | 0.07 ± 0.37 | 0.04 ± 0.28 | 0.669 |
| Operations on the Ear | 0 ± 0.06 | 0 ± 0.05 | - |
| Operations on the Nose, Mouth, and Pharynx | 0.02 ± 0.2 | 0.02 ± 0.2 | 0.740 |
| Operations on the Respiratory System | 0.11 ± 0.51 | 0.08 ± 0.45 | 0.745 |
| Operations on the Cardiovascular System | 0.17 ± 0.73 | 0.13 ± 0.58 | 0.840 |
| Operations on the Hemic and Lymphatic System | 0.04 ± 0.23 | 0.03 ± 0.2 | 0.805 |
| Operations on the Digestive System | 0.26 ± 1.07 | 0.22 ± 0.93 | 0.847 |
| Operations on the Urinary System | 0.12 ± 0.67 | 0.09 ± 0.53 | 0.952 |
| Operations on the Male Genital Organs | 0.01 ± 0.16 | 0.01 ± 0.14 | - |
| Operations on the Female Genital Organs | 0.03 ± 0.27 | 0.02 ± 0.22 | 0.944 |
| Operations on the Musculoskeletal System | 0.1 ± 0.46 | 0.07 ± 0.37 | 0.719 |
| Operations on the Integumentary System | 0.04 ± 0.3 | 0.03 ± 0.26 | 0.647 |
| Obstetrical Procedures | 0 ± 0.01 | 0 ± 0.02 | - |
| Miscellaneous Diagnostic and Therapeutic Procedures | 2.27 ± 3.88 | 1.35 ± 2.7 | **0.015** |

**Supplementary Table 3.** Prescribed drugs in COVID-19 context. Mean ± SD. p-value of test of difference in means of COVID-19 positive vs negative individuals.

| ***Drugs*** | **COVID+** | **COVID-** | ***p*-value** |
| --- | --- | --- | --- |
| Alimentary Tract and Metabolism Drugs | 2.56 ± 3.23 | 1.88 ± 2.65 | 0.068 |
| Blood and Blood Forming Organs Drugs | 1.32 ± 1.97 | 0.86 ± 1.53 | **0.036** |
| Cardiovascular System Drugs | 1.86 ± 2.83 | 1.36 ± 2.28 | 0.164 |
| Dermatologicals | 0.94 ± 2.15 | 0.53 ± 1.43 | 0.054 |
| Genitourinary System and Sex Hormones Drugs | 0.18 ± 0.66 | 0.11 ± 0.47 | 0.430 |
| Systemic Hormonal Preparations, Excluding Sex Hormones and Insulins | 1.17 ± 2.07 | 0.8 ± 1.62 | 0.078 |
| Antiinfectives for Systemic Use | 2.97 ± 3.67 | 1.83 ± 2.74 | **0.001** |
| Antineoplastic and Immunomodulating Drugs | 0.1 ± 0.56 | 0.08 ± 0.44 | 0.805 |
| Musculoskeletal System Drugs | 0.48 ± 1.1 | 0.36 ± 0.91 | 0.446 |
| Nervous System Drugs | 5.69 ± 6.28 | 4.23 ± 5.39 | 0.052 |
| Antiparasitic Products, Insecticides, and Repellents | 0.03 ± 0.23 | 0.02 ± 0.19 | - |
| Respiratory System Drugs | 1.19 ± 2.27 | 0.72 ± 1.71 | 0.069 |
| Sensory Organs Drugs | 0.64 ± 1.6 | 0.35 ± 1.15 | 0.068 |
| Various Drugs | 0.32 ± 0.77 | 0.24 ± 0.69 | 0.431 |
| Total Number of Drugs | 19.45 ± 16.32 | 13.36 ± 12.81 | **< 0.001** |

|  | **WOMEN** | | | **MEN** | | |
| --- | --- | --- | --- | --- | --- | --- |
| ***Use of resources*** | **COVID+** | **COVID-** | ***p*-value** | **COVID+** | **COVID-** | ***p*-value** |
| Primary Care Nurse | 23.63 ± 29.49 | 18.15 ± 26.35 | **0.037** | 20.66 ± 26.7 | 14.51 ± 21.8 | **0.003** |
| Primary Care Physician | 24 ± 20.41 | 17.48 ± 16.62 | **0.002** | 21.99 ± 18.93 | 15.47 ± 15.41 | **< 0.001** |
| Outpatients | 7.84 ± 14.93 | 5.94 ± 12.34 | 0.344 | 10.62 ± 16.53 | 8.43 ± 13.81 | 0.381 |
| Home hospitalizations | 2.62 ± 8.03 | 2.28 ± 7.05 | 0.845 | 3.46 ± 11.46 | 2.7 ± 8.47 | 0.841 |
| Hospitalizations | 20.34 ± 22.7 | 12.83 ± 17.09 | **< 0.001** | 28.01 ± 29.58 | 17.78 ± 22.67 | **< 0.001** |
| ICU | 0.07 ± 0.29 | 0.04 ± 0.2 | 0.314 | 0.15 ± 0.39 | 0.07 ± 0.27 | **0.023** |
| Days at ICU | 0.96 ± 5.25 | 0.23 ± 2.42 | 0.352 | 2.76 ± 10.62 | 0.46 ± 3.83 | **0.017** |
| Emergencies | 3.41 ± 3.56 | 2.12 ± 2.79 | **< 0.001** | 4.06 ± 4.68 | 2.49 ± 3.26 | **< 0.001** |

**Supplementary Table 4.** Use of resources in COVID-19 context divided by sex. Mean ± SD. *p*-value of test of difference in means of COVID-19 positive vs negative individuals for each sex.

**Supplementary Figure 1**


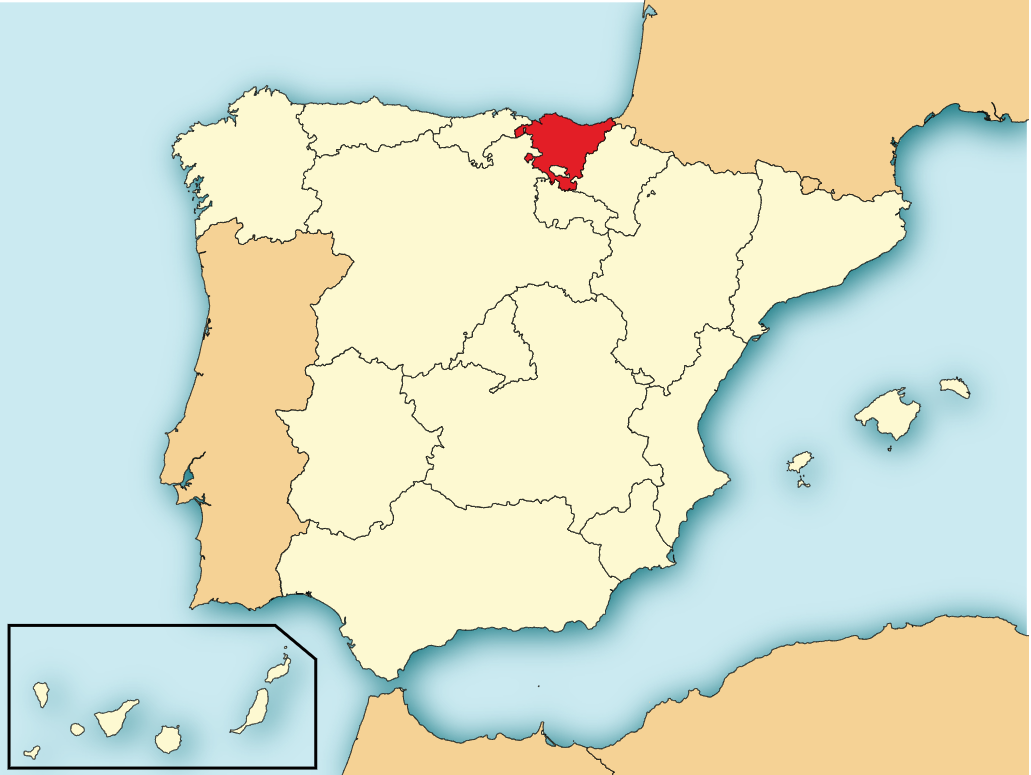


**A**

**B**

Individuals after data curation (2020-2022)

(n = 22,251)

Individuals registered in the database (2020-2022)

(n = 22,418)

Individuals included in the analysis

(n = 21,495)

Poor quality or missing data

(n = 167)

Individuals deceased before pandemic period

(n = 756)

**Figure legend. A)** Workflow of data cleansing for the study cohort. **B)** Location of the Basque Country in Spain. Image under Creative Commons License.

Source: https://es.m.wikipedia.org/wiki/Archivo:Localizaci%C3%B3n_del_Pa%C3%ADs_Vasco.svg#file

**Supplementary Figure 2**


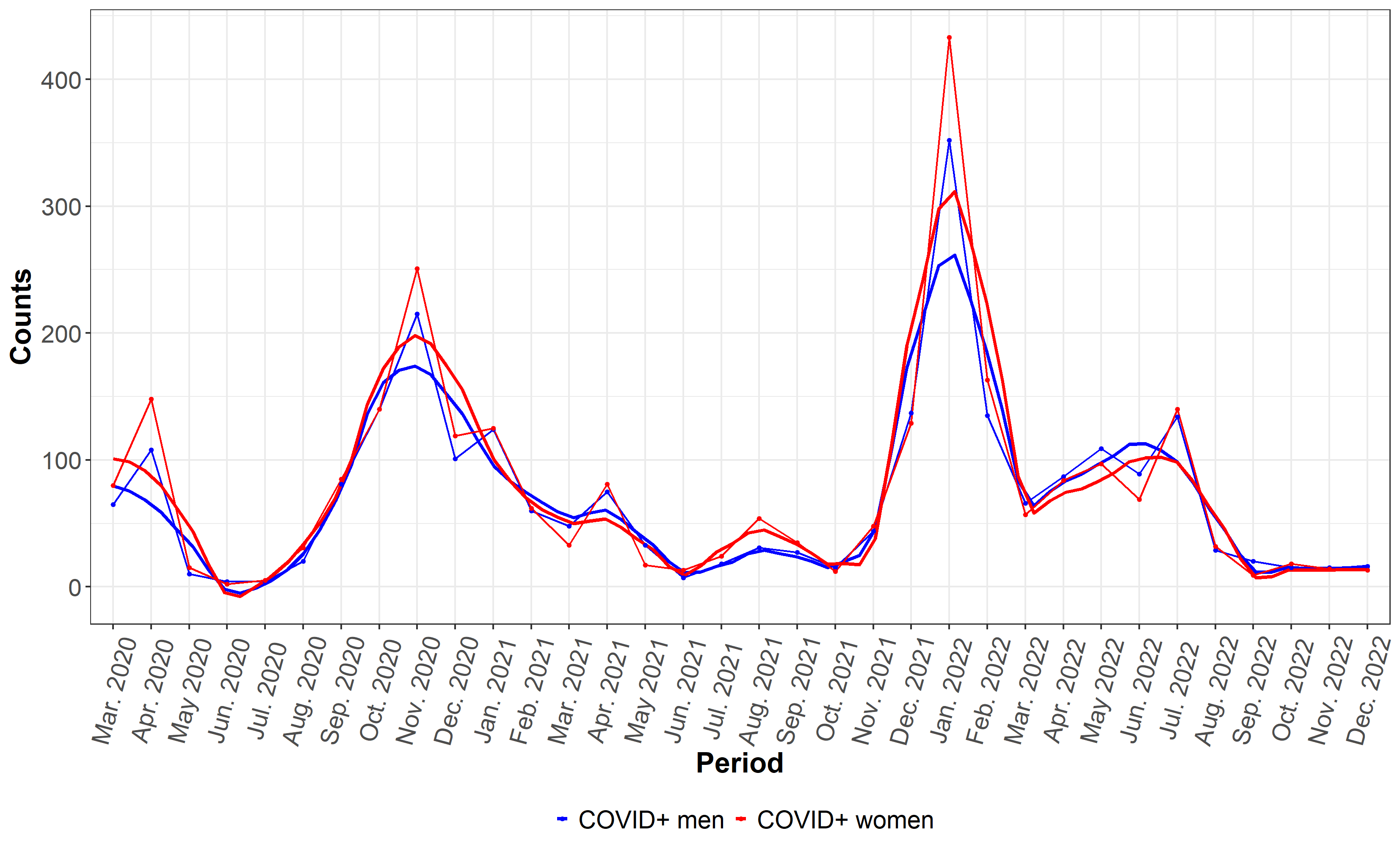

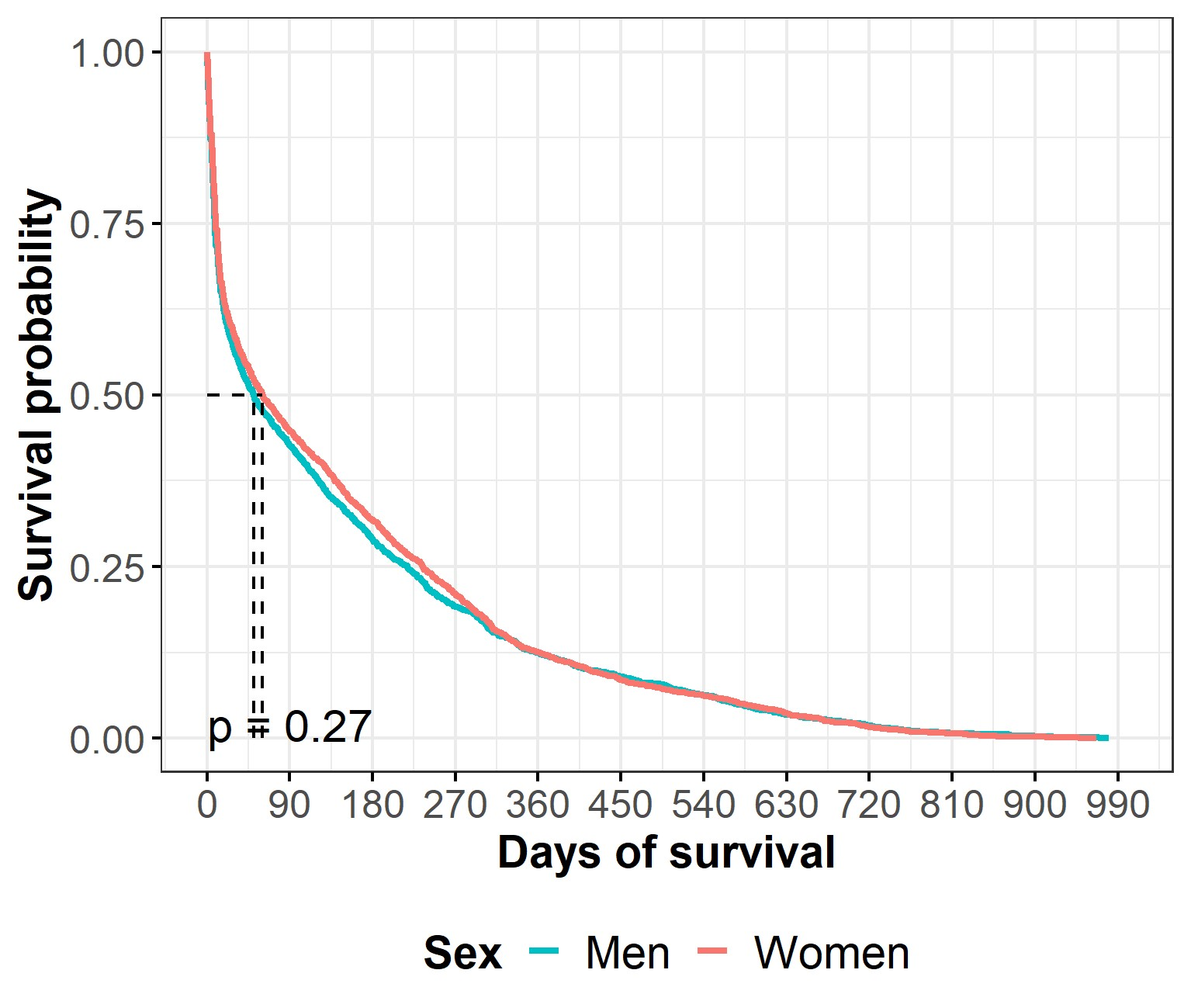


**B**

**C**


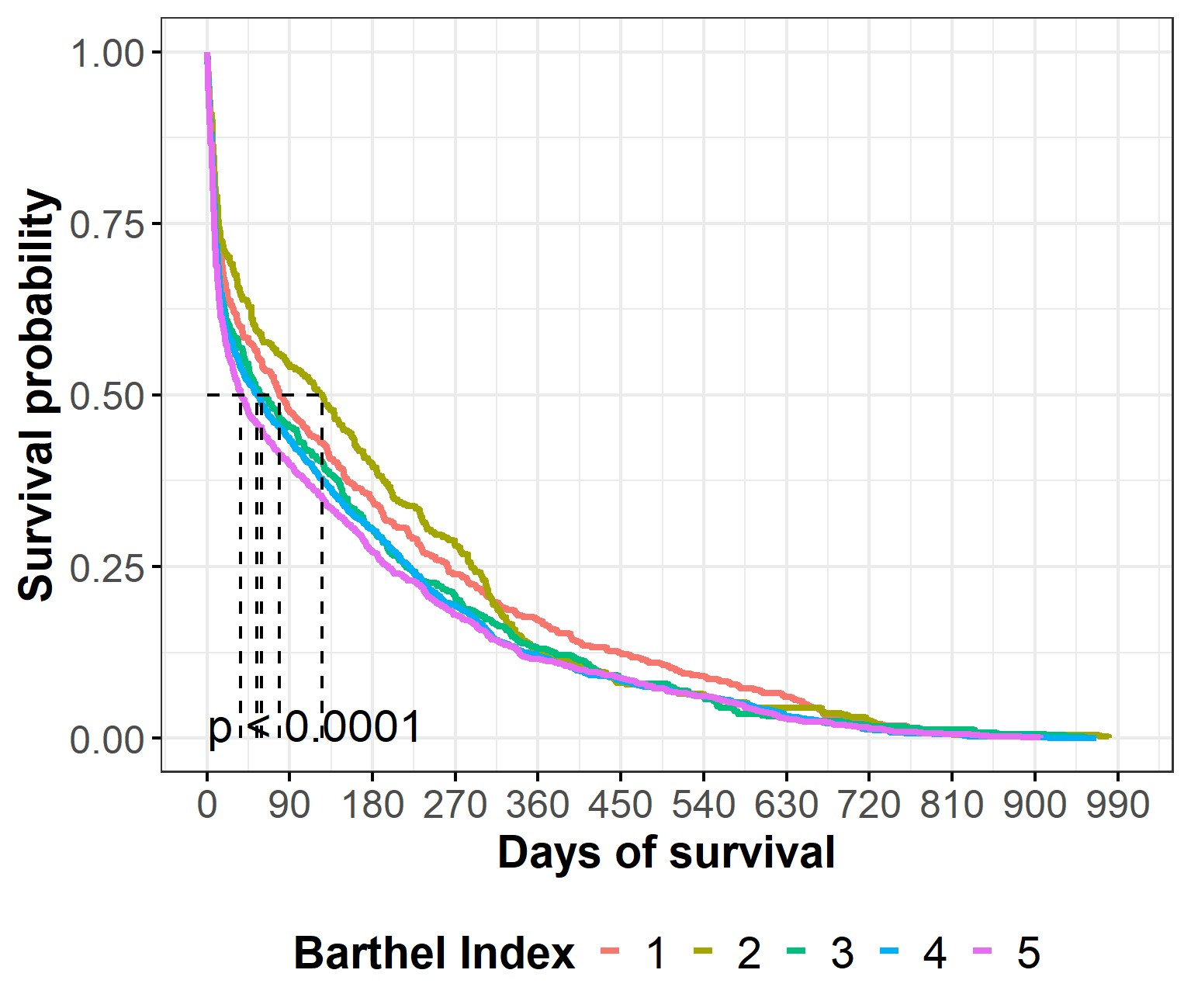


**A**

**Figure legend. A)** Evolution of COVID-19-positive cases divided by sex. **B)** Overall survival of COVID-19-positive men vs women. **C)** Overall survival of COVID-19-positive cases in function of Barthel Index, from 1 (totally dependent) to 5 (independent).
