# Supplementary material for: Electronic health records reveal that COVID-19 impacted health resources and survival of Basque population
Source: Aging Clin Exp Res. 2024 Nov 29;36(1):228. doi: 10.1007/s40520-024-02884-7 (PMC11606984; doi:10.1007/s40520-024-02884-7)
Supplement: Supplementary file 2 — Supplementary file2 (PPTX 3104 KB) [file 40520_2024_2884_MOESM2_ESM.pptx]

## Slide 1
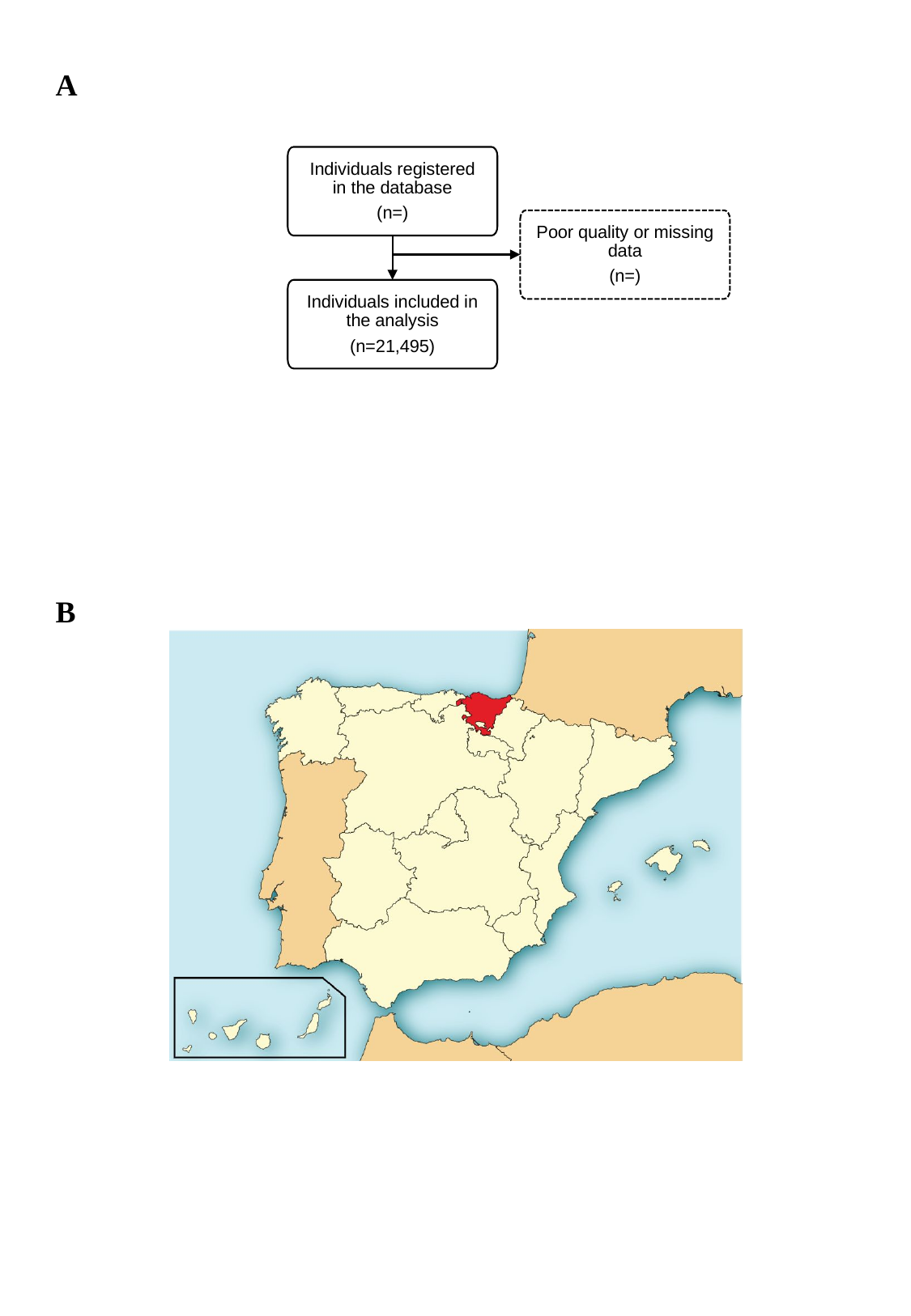

A
Individuals registered in the database
(n=)
Individuals included in the analysis
(n=21,495)
Poor quality or missing data
(n=)
B

## Slide 2
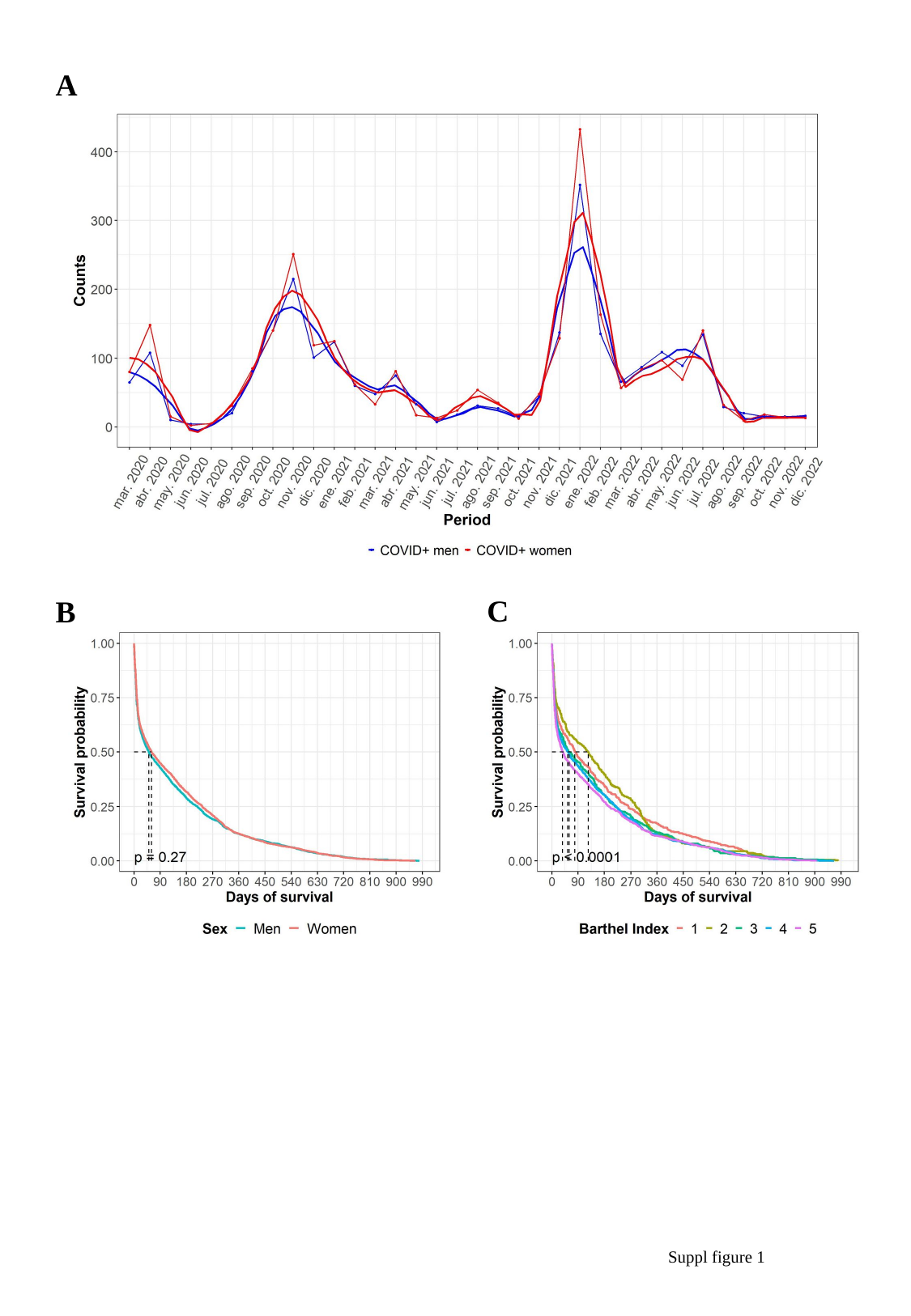

A
C
B
Suppl figure 1
